# Global Distribution and Diversity of Haloarchaeal pL6-Family Plasmids

**DOI:** 10.3390/genes15091123

**Published:** 2024-08-26

**Authors:** Mike Dyall-Smith, Friedhelm Pfeiffer

**Affiliations:** 1 Veterinary Biosciences, Melbourne Veterinary School, Faculty of Science, University of Melbourne, Parkville, VIC 3010, Australia; mike.dyallsmith@gmail.com; 2 Computational Systems Biochemistry, Max-Planck-Institute of Biochemistry, 82152 Martinsried, Germany

**Keywords:** plasmid, haloarchaea, halobacteria, *Haloquadratum*, pleolipovirus, salt lake, saltern, crystallizer pond

## Abstract

Australian isolates of *Haloquadratum walsbyi*, a square-shaped haloarchaeon, often harbor small cryptic plasmids of the pL6-family, approximately 6 kb in size, and five examples have been previously described. These plasmids exhibit a highly conserved gene arrangement and encode replicases similar to those of betapleolipoviruses. To assess their global distribution and recover more examples for analysis, fifteen additional plasmids were reconstructed from the metagenomes of seven hypersaline sites across four countries: Argentina, Australia, Puerto Rico, and Spain. Including the five previously described plasmids, the average plasmid size is 6002 bp, with an average G+C content of 52.5%. The tetramers GGCC and CTAG are either absent or significantly under-represented, except in the two plasmids with the highest %G+C. All plasmids share a similar arrangement of genes organized as outwardly facing replication and ATPase modules, but variations were observed in some core genes, such as F2, and some plasmids had acquired accessory genes. Two plasmids, pCOLO-c1 and pISLA-c6, shared 92.7% nt identity despite originating from Argentina and Spain, respectively. Numerous metagenomic CRISPR spacers matched sequences in the fifteen reconstructed plasmids, indicating frequent invasion of haloarchaea. Spacers could be assigned to haloarchaeal genera by mapping their associated direct repeats (DR), with half of these matching *Haloquadratum*. Finally, strand-specific metatranscriptome (RNA-seq) data could be used to demonstrate the active transcription of two pL6-family plasmids, including antisense transcripts.

## 1. Introduction

*H. walsbyi* is an extremely halophilic archaeon (class *Halobacteria*) with cells that are thin squares. It thrives in environments with salt concentrations near saturation, such as salt lakes and saltern crystallizer ponds [1,2,3,4,5]. Although slow growing and relatively difficult to culture, *Hqr. walsbyi* often reaches community dominance and is the main cell type (≥50%) observed by microscopy [5,6,7] or detected by sequence analysis [8,9,10,11]. Its high community abundance has been correlated with elevated magnesium concentrations [1,2,12]. Due to its poor growth on agar plates, making plaque assays impossible, viruses of *Hqr. walsbyi* have been studied by metagenomics [13,14,15,16] rather than by virus isolation.

**Figure 1:**
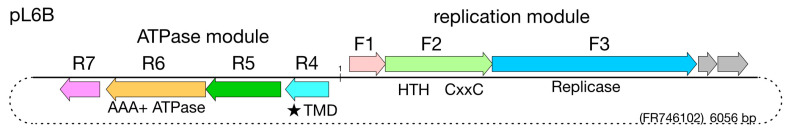
Plasmid pL6B gene diagram showing the three core forward genes of the replication module (F1 to F3) and the four core genes of the leftward ATPase module (R4 to R7). The plasmid start base (nt 1), shown in the center, is set between R4 and F1. HTH, helix-turn-helix domain; CxxC, Cys-x-x-Cys motif; (star) TMD, transmembrane domain. The dotted line indicates circularity. Sequence accession and plasmid size are shown in the lower right.

A curious feature of the genome of *Hqr. walsbyi* strain C23^T^ was the discovery of two small, circular plasmids (pL6A and pL6B) that were of similar size (6056 and 6129 bp) and gene content and shared 76% sequence identity [4]. Additional Australian isolates of *Hqr. walsbyi* were also found to carry pL6-related plasmids [17], indicating that they were common in this genus. In an earlier metagenomic study of Lake Tyrrell, a hypersaline lake, Tully et al. found that plasmids similar to pL6B were present in at least 32–40% of the *Haloquadratum* population [9]. These plasmids were so common that the metagenomic data from Lake Tyrrell contained sufficient reads to be able to reconstruct a pL6-like plasmid, pLTMV-6 [17]. Only five pL6-family plasmids have so far been described, and all share seven core genes with a conserved synteny, a size range of 5.8–7.0 kb, and a GC content (51–53%) significantly higher than that of the chromosome of *Haloquadratum* (48%). A diagram of pL6B (Figure 1) illustrates the seven core genes arranged in two outwardly oriented modules. The replication module has forward genes F1 to F3, and the ATPase module has four reverse-oriented genes R4 to R7. Only the R4 protein (Figure 1) has been experimentally detected in the proteome of *Hqr. walsbyi* [4], where it was associated with a membrane fraction, consistent with the presence of a C-terminal transmembrane domain (TMD; asterisked in Figure 1).

At the nucleotide level, these replicons are 61–79% identical. They lack integrase genes and have only been found as episomal circular plasmids. None carry a DNA polymerase gene, but the F3 gene has been proposed to encode a replicase [18,19]. In the best-studied case of *Hqr. walsbyi* strain C23^T^, these plasmids are multi-copy, with a copy number of pL6A and pL6B (combined) estimated as ~30 copies/genome equivalent [4]. It remains unclear how pL6A and pL6B are both maintained in the host cell population.

Two of the core genes of these plasmids (F3 and R6) specify proteins similar to those of haloviruses. The F3 protein of pL6B is 45% identical to the replicase protein ORF9 (HRPV-3_gp09) of Halorubrum pleomorphic virus 3 (HRPV-3), a betapleolipovirus [18,19]. R6 is a predicted AAA+ ATPase, and the pL6B protein shows 24% identity to the ATPase (His1V_gp16) of salterprovirus His1 [20,21], a lemon-shaped virus currently classified in the family *Halspiviridae* [22]. His1V_gp16 is most likely the virus packaging ATPase [23].

It is often difficult to distinguish plasmids from temperate viruses if the capsid proteins are novel, such as in the case of pHK2, originally thought to be a plasmid [24] but later found to be a temperate pleolipovirus [25]. The circumstantial evidence that pL6-family plasmids may represent a novel group of temperate viruses can be summarized in three points, (a) they carry two genes that encode proteins related to known haloviruses [17], (b) the seven core genes display a highly conserved synteny and sequence conservation, and (c) they can be found in metaviromic sequence data [26], with the caveat that these post-0.1 μm filtrate metagenomes probably also include DNA from extracellular vesicles (EV) [27,28,29]. The previously described pL6 elements vary in size from 5 to 7 kb, a range that overlaps at the top end with the smallest pleolipovirus genome (HRPV1, 7 kb), while the lower end is close to the 5278 bp genome of Aeropyrum pernix bacilliform virus 1 (APBV1), a rod-shaped archaeal virus [30]. More generally, bacterial viruses of the *Microviridae* family have DNA genomes of 5 kb or less [31].

In this study, publicly available metagenomic data from hypersaline lakes and saltern crystallizer ponds were used to reconstruct plasmids related to the known members of the pL6 family. Their annotation and comparison revealed greater diversity in gene content and arrangement than previously recognized. These plasmids were found to match numerous CRISPR spacers retrieved from the same metagenome, as well as those present in metagenomes of other hypersaline sites. In addition, recently available RNA-seq data from the microbial communities of two sites (Lake Tyrrell and Santa Pola) contained numerous reads mapping to both strands of autochthonous pL6-family plasmids, demonstrating active transcription and significant levels of counter-transcripts.

## 2. Materials and Methods

### 2.1. Metagenomic Sequence Data and Plasmid Assembly Using metaplasmidSpades

Metagenomes with *H. walsbyi* were identified in the SRA database using the Branchwater server (https://branchwater.jgi.doe.gov/; accessed 25 January 2024) and the *Hqr. walsbyi* C23 genome sequence (FR746099.1) as the query sequence. This returned 77 metagenomes with containment-based average nucleotide identity (cANI) scores of 1. These were only subjected to more detailed analysis when the read length was (a) at least 150 nt and reads were paired or (b) the average read length was >500 nt. Candidate metagenomes were individually examined for reads matching pL6-family plasmids using the SRA BLASTn option available on each SRA web page, and those showing numerous matches (>100) were selected for plasmid reconstruction attempts. Metagenomes from which pL6-like plasmids could be reconstructed are described in Table S1. Fastq reads were downloaded from the ENA website (https://www.ebi.ac.uk/ena/browser/home, accessed on 15 January 2024), imported into Geneious Prime v. 2023.2.1 (https://www.geneious.com), and trimmed using BBDuk (v. 38.84). Trimmed reads were exported as fastq reads and uploaded to a Galaxy server (https://usegalaxy.org.au) as input for metaplasmidSpades (with default settings). Circular contigs assembled by metaplasmidSpades [32] were then imported back into Geneious for further analysis.

### 2.2. Manual Assembly of pL6-Related Plasmids from Metagenomes

This is based on the read-extension method described earlier for reconstructing the plasmid pLTMV-6 [17]. Trimmed sequence reads were first mapped to all known pL6-family plasmid sequences using parameters that relax the mapping stringency (Geneious mapper with the following settings: custom sensitivity, fine-tuning, none(fast/read mapping), max mismatches/read = 35%, max ambiguity = 5, allow gaps (2%), max gap size = 6). Reads matching the F3 genes were extracted, de novo assembled using stringent assembly parameters (Geneious assembler, custom sensitivity, max mismatches per read = 2%, max ambiguity = 2), and the resulting contigs examined for sequences of high confidence and read coverage (≥10×). A region (400–1200 nt) was then selected and extended at both ends by repeated rounds of read mapping using the full set of metagenomic (paired) reads with the same stringent assembly parameters as those used for the de novo assembly. After each mapping round, the contig termini were examined for paired reads that overlapped the contig ends by at least 100 nt. In this manner, contig extension proceeded with high confidence. Consecutive cycles of mapping and contig extension were performed until the sequence was repeating at the termini, indicating that the borders of the plasmid had been crossed and that ring closure had been achieved. Depending on the coverage, read length, and distance between read pairs, this process required up to 37 rounds. A monomeric sequence was generated by trimming the terminal duplication. Subsequently, the first base was set to a point as similar as possible to that of pL6A (accession FR746101), and the genes were annotated using a combination of similarity to previously annotated pL6 plasmids, assisted by annotation tools including GeneMarkS-2 [33] and phanotate [34].

### 2.3. Plasmid pTYRR-r1

As a starting point, the RNA-seq data of the 2018 microbial community of Lake Tyrrell (SRR24125903) [10] was mapped to all pL6-family plasmids using the Geneious Prime ‘map to reference’ tool (threshold of ≤2% mismatches) and by far the most reads matched to pLTMV-6 (367 reads), a plasmid reconstructed from a Lake Tyrrell metagenome and described previously [17]. In the next stage, an initial seed contig was generated from RNA-seq reads (SRR24125903-4) mapped at relaxed stringency (same settings as for the other plasmids) to the F3 gene of pLTMV-6. The mapped reads were de novo assembled with stringent assembly parameters (as used for the other plasmids), and a 400 nt region of this contig was selected based on its high confidence and read coverage. In the final stage, metaviromic DNA reads from Lake Tyrrell viral concentrates (SRR5637210, SRR5637211, SRR402042, and SRR402044) were used to extend the RNA-seq-based seed sequence through repeated rounds of read mapping (conditions were the same as above) until ring closure. This produced a plasmid, pTYRR-r1, which was expected to be more representative of plasmids occurring at the time of sampling used in the RNA-seq study [10].

### 2.4. CRISPR Spacer Searches

Sequences from existing and newly generated plasmids were used to search for matching spacers using the IMG/VR Viral/Spacer BLAST server (https://img.jgi.doe.gov/cgi-bin/vr/main.cgi, accessed on 1 March 2024). In addition, spacers were retrieved from metagenomes using the MinCED tool (https://github.com/ctSkennerton/minced, accessed on 15 January 2024). The spacers were then imported into Geneious and formatted as a BLASTn database. Plasmid sequences were used to query the spacer database, and the best hits were inspected to check whether their flanking direct repeats (DR) were similar to known CRISPR arrays using the CRISPR-Cas++ database (https://crisprcas.i2bc.paris-saclay.fr, accessed on 15 January, 2024) and BLASTn searches at NCBI (https://blast.ncbi.nlm.nih.gov/, accessed on 15 January, 2024) against the WGS database (Expect threshold, 10^−3^; Organism: Archaea).

### 2.5. RNA-Seq Data Used for Mapping to the pL6 Plasmids pTYRR-r1 and pPOLA-c1

As with the metagenomic data, RNA-seq data (see Table S1) were downloaded from ENA (https://www.ebi.ac.uk/ena/browser/home, accessed on 10 March 2024) and imported into Geneious Prime (v. 2023.2.1), and the ends were trimmed of low-quality bases using BBDuk (v. 38.84), available in the Geneious environment. Ribosomal RNA reads were depleted by mapping to the ribosomal RNA operon from *Hqr. walsbyi* C23 (NC_017459; nt 66,889–72,015) using the Geneious ‘map to reference’ tool. Trimmed unmapped reads were mapped to plasmid sequences derived from the same hypersaline site (pTYRR-r1/Lake Tyrrell; pPOLA-c1/Santa Pola) using the ‘map to reference’ tool within Geneious Prime (settings: custom sensitivity, fine-tuning = none (fast/read mapping); max mismatches per read = 1%, max ambiguity = 1; no gaps).

### 2.6. Phylogenetic Tree Reconstruction

Complete nucleotide sequences of all pL6-family plasmids were aligned using the MAFFT (v7.490) aligner [35] in Geneious Prime (ver. 2024.0.5), and this was used to infer a consensus tree using Geneious Tree Builder, the Neighbor-Joining method, and 1000 bootstrap repetitions.

Proteins similar to the F3 or R6 proteins of the pL6-family plasmids were retrieved from the GenBank nr protein database using BLASTp (blast.ncbi.nlm.nih.gov/Blast.cgi, accessed on 26 April 2024). For F3 homologs, only those that could be aligned with ≥90% of the query sequence (highest E-value, 2 × 10^−54^) were selected for tree reconstruction. For R6-related proteins, only relatively few could be retrieved, and homologs with a query coverage of ≥70% were selected. The F3 proteins of the newly reconstructed pL6 plasmids were added to the proteins retrieved from GenBank, aligned using the COBALT aligner (www.ncbi.nlm.nih.gov/tools/cobalt/cobalt.cgi, accessed on 28 April 2024) with default parameters, and then imported into Geneious Prime where a Neighbor-Joining consensus tree (100 bootstrap repetitions) was generated using the Geneious Tree Builder tool. The R6-related proteins were aligned using MAFFT within Geneious Prime, and then a consensus tree was inferred using the Neighbor-Joining method (within Geneious Prime) and 1000 bootstrap replications.

### 2.7. Other Bioinformatics Tools

Nucleotide and protein sequence alignments were performed in the Geneious Prime (v. 2023.2.1) environment using MUSCLE (v. 5.1) or MAFFT (v7.490) aligners, using default settings. Protein domain searches were performed using InterProScan (https://www.ebi.ac.uk/interpro/result/InterProScan, accessed on 1 February 2024) and the conserved domains server (https://www.ncbi.nlm.nih.gov/Structure/cdd/wrpsb.cgi, accessed on 10 February 2024). Helix-turn-helix (HTH) motifs were detected using (https://users.cis.fiu.edu/~giri/bioinf/GYM2/prog.html, accessed on 20 February 2024) and (https://npsa.lyon.inserm.fr/cgi-bin/npsa_automat.pl?page=/NPSA/npsa_hth.html, accessed on 20 February 2024). Tetramer analysis of DNA was performed with (http://gscompare.ehu.eus/tools/oligo_frequencies/, accessed on 23 April 2024). Alphafold 2 structure predictions used Galaxy Version 2.3.1 of this software, available on the Galaxy server at https://usegalaxy.org.au (accessed on 10 May 2024). Protein structure similarity searches were performed using Foldseek (https://search.foldseek.com, accessed on 15 May 2024).

## 3. Results

### 3.1. Assembly of pL6-Family Plasmids from Publicly Available Metagenomes

**Figure 2:**
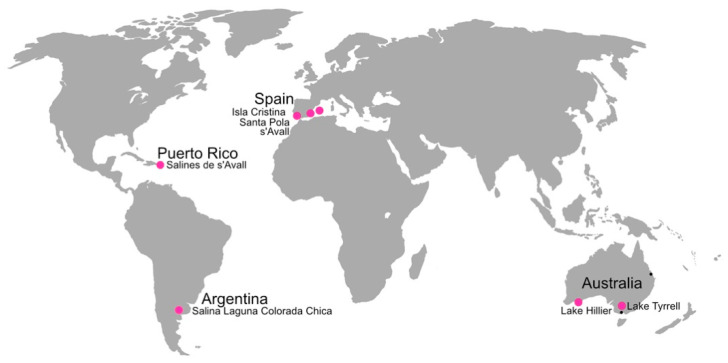
Global map of the hypersaline sites from which metagenomic data were used to reconstruct pL6-family plasmids (large pink spots). In addition, two coastal Australian sites, where *Hqr. walsbyi* strains carrying pL6-family plasmids were previously isolated are indicated by black dots.

The metagenomes of the hypersaline waters used to reconstruct the pL6-like plasmids are listed in Table S1, and their sample locations are indicated on the world map shown in Figure 2. These metagenomes were selected based on the abundance of *Hqr. walsbyi* and high numbers of reads matching the pL6-family plasmids (see Section 2). The only exceptions were metavirome sequences, which contained relatively low levels of cellular DNA. Reconstruction of pL6-family plasmids from metagenome (or metavirome) reads followed two strategies. The first used multiple cycles of read extension from an initial seed sequence and was previously described for the reconstruction of the plasmid pLTMV-6 [17]. The second method used metaplasmidSpades, an automated pipeline [32] that assembles plasmids from metagenomic reads.

Fifteen novel pL6-family plasmids were reconstructed, and their general features are given in Table 1, along with the five previously reported pL6-family plasmids [17]. Details regarding read coverage are provided in Table S2 and Figure S1 (panels A–O). For convenience, all twenty plasmids will be used in the following comparisons.

Plasmid sizes range from 5.0 to 7.6 kb (average of 6002 bp), and their %G+C values vary from 47.8 to 64.0 (average 52.5%, median 52.2%), but two of the new plasmids are outliers with significantly higher values than the other 18 which have %G+C values below 54%. These are pCABO-s5 (58.5%) and pISLA-s1 (64.0%). These outliers show a number of divergent features (see later) and are referred to as high-GC plasmids. Most genera within the class *Halobacteria* have genomes with high %G+C values, ranging from 61 to 70% (see Table S4 of [36]), but *Hqr. walsbyi* is atypical in having a much lower %G+C, averaging 47.8% [4].

DNA sequence alignment of all 20 plasmids gave an average pairwise nucleotide identity of 47.7% (range 28.5–92.7%). The two most closely related plasmids are pCOLO-c1 and pISLA-c6, which show 92.7% nt identity, a surprisingly high value given the geographical distance between their sites of origin (Argentina and Spain, respectively).

Tetramer frequency analysis of plasmid sequences (Table S3) identified two motifs, GGCC and CTAG, with significant under-representation. GGCC is absent in 17 of the 20 plasmids and strongly under-represented in pBAJ9-6, which has only two closely spaced motifs located between the F3 and R7 genes, a common region for foreign gene acquisition (see later). However, GGCC was not under-represented in the two high GC plasmids (pCABO-s5 and pISLA-s1), indicating that this site is not under selection in their normal host species.

CTAG is absent in 10 plasmids, under-represented in 7, and present at expected levels in the two high GC plasmids (see Table S2). The remaining plasmid, pHILL-c2, was unusual in having the expected level of CTAG motifs (8 sites), but four of these sites are found between the F3 and R7 genes, all within a 122 bp region (nt 3182–3303) near an acquired gene encoding a formyltransferase-family protein. It is likely that the region containing these sites was also acquired, and outside this region, the motif is also under-represented (0.57, second-order Markov chain estimate). Finally, the tetramer TTAA was found to be absent in pISLA-s1 but present in all other plasmids.

Selection against longer motifs is more difficult to assess given the small size of pL6-family members, but the 6-mer GGATCC is absent in all plasmids except pTYRR-r1, where it occurs only once within an acquired gene encoding a helicase located between the F3 and R7 genes.

The results suggest that the two high GC plasmids originate from haloarchaea that do not select against CTAG and GGCC motifs and possibly a species with a higher %G+C than *Hqr. walsbyi*.

**Figure 3:**
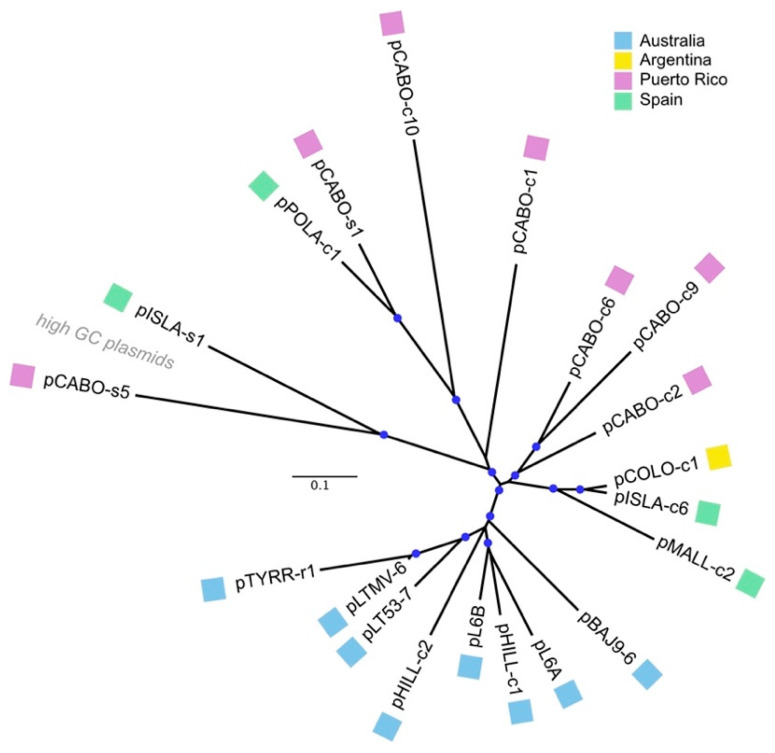
Phylogenetic tree reconstruction of pL6-family plasmids using complete nucleotide sequences (see Section 2). Sequences were aligned using MAFFT, and a Neighbor-Joining consensus tree generated using Geneious Tree Builder. The scale bar represents the inferred changes per site. Blue circles at branch points indicate bootstrap confidence values of 96–100% (1000 bootstrap repetitions). Colored boxes next to plasmid names show the country of origin (key at the top right).

Figure 3 shows an unrooted phylogenetic tree inferred from the complete sequences of the pL6-family plasmids (see Section 2). The bootstrap confidence values at most branch points are above 95% (from 1000 repetitions). The eight plasmids from Australian sites all branched together, forming a single clade. The two high GC plasmids (left) from Spain and Puerto Rico formed a distinct clade, but with long branch lengths between them. pCABO-s1 (Puerto Rico) and pPOLA-c1 (Spain) also branch closely together. The other eight plasmids show a mixture of relationships between plasmids from different sites; for example, plasmids pCOLO-c1 (Argentina) and pISLA-c6 (mainland Spain) branched very closely together, and they form a sister group with pMALL-c2, from the Mediterranean island of Mallorca.

### 3.2. Relative Presence of Plasmid pPOLA-c1 in Fractionated Samples from Santa Pola Crystallizer

In the study of [11], saltern crystallizer water samples from Santa Pola, Spain, were fractionated using various combinations of filtration and centrifugation to purify dissolved DNA and viruses. These brines were rich in *Haloquadratum*, with an abundance estimated at 63% of prokaryotic genera. Since pPOLA-c1 was reconstructed from the reads of that study (Table 1), it could be used to probe the relative levels of this plasmid in each fraction. Equal numbers of paired reads (3 million) from the four fractions were mapped to the pPOLA-c1 sequence, and the number of matching reads for each is given in Table 2. Compared to the DNA of the cell pellet (iDNA), pPOLA-c1 was present at raised levels (1.6–2.6-fold) in dissolved DNA produced by the two methods (FdDNA, CdDNA). However, there were few matching reads in the ‘virus DNA’ preparation obtained from pelleted material collected after ultracentrifugation of cell-free fractions, suggesting pPOLA-c1 is only present as dissolved DNA. Various interpretations of these results can be found in Section 4.

### 3.3. Comparative Gene Maps of pL6-Family Plasmids

**Figure 4:**
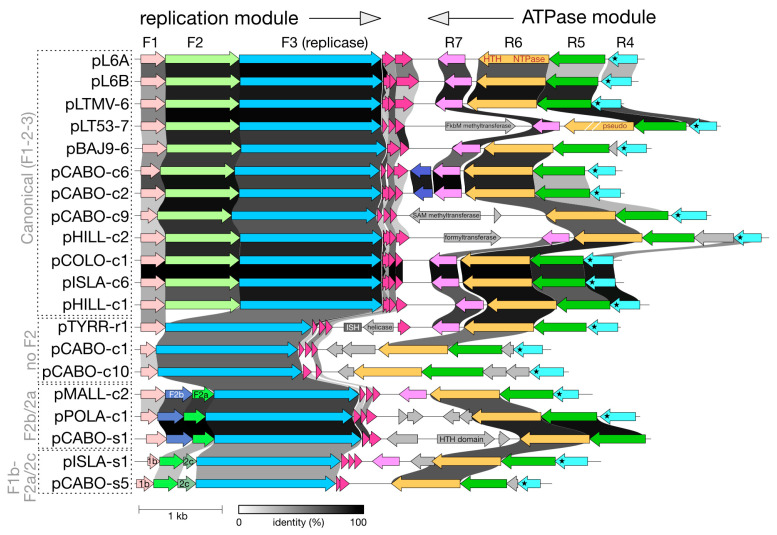
Gene maps of all pL6-family members. Plasmids are named at the left edge and have been linearized such that the starting base is leftmost. Boxed plasmid names and gray labels on the left indicate the grouping of plasmids based on similarities in their replication modules (see text). Annotated genes are either color-coded to indicate corresponding homologs or gray shaded to indicate that they do not have homologs in other pL6 plasmids. The violet-colored accessory genes of pCABO-c6 and pCABO-c2 (between F3 and R7) encode similar hypothetical proteins. The previously used nomenclature for the main proteins (F1-3 and R4-7) is shown at the top. R4-proteins all have a predicted transmembrane domain (indicated by asterisks) at their C-termini. Grayscale shading between CDS indicates nucleotide similarity (see identity (%) key underneath). The scale bar (1 kb) is shown below.

The reconstructed plasmids were annotated (see Section 2), and their gene maps were compared to the five previously described pL6-family members (Figure 4). Plasmids are shown linearized and start at base 1 on the left. Gray shading between corresponding genes of each map indicates their nucleotide similarity, and related CDS have been color-coded to highlight relationships. For ease of comparison, the twenty plasmids have been clustered into four groups according to the similarity of their replication modules. It is clear from Figure 4 that pL6-family plasmids share a common pattern of gene organization, although several of the newly reconstructed representatives show variations in this basic pattern, which are described below. A summary of gene variants is given in Table 3, and alignments of plasmid-encoded proteins are provided as supplementary figures (Figures S2, panels A–I) to aid these descriptions. Further details regarding the core proteins are provided in Supplementary Text S1.

#### 3.3.1. Replication (Forward) Module

In all plasmids, the genes from F1 to F3 in this module overlap at stop/start codons, indicating a single transcriptional unit that is probably driven by a promoter within the R4-F1 intergenic region (Figure 1, and see later). Most plasmids (12/20) fall into the ‘canonical group’ (Figure 4, Table 3) and carry full-length versions of the three canonical forward genes F1-F2-F3, as found in pL6A. This group includes plasmids from Australia, Argentina, Puerto Rico, and Spain. Deviating from this pattern are three groups of plasmids that are boxed and labeled at the left edge of the figure. The first of these, labeled ‘no F2’, consists of three plasmids that have lost the F2 gene entirely (pTYRR-r1, pCABO-c1, and pCABO-c10), indicating that this gene is either dispensable or the plasmids are defective. The next group (F2b/2a) of three plasmids (pMALL-c2, pPOLA-c1, and pCABO-s1) replaced F2 with two smaller CDS, labeled F2b and F2a. F2a shows sequence similarity to the N-terminal region of F2 and a structural relationship (Supplementary Text S1a and Figure S3). F2b is unrelated to F2. The third group (F1b/F2a/F2c) consists of the high GC plasmids pISLA-s1 and pCABO-s5, where each carries a divergent F1 gene (F1b), followed by F2a and a novel gene (F2c) before F3. F2c is unrelated to F2. In all plasmids, a number of small CDS occur just after F3 and in the same orientation (colored crimson in Figure 4) but they will not be discussed further because they are very short and their annotation remains uncertain. More detailed descriptions of the replication module genes and proteins are provided in Supplementary Text S1a.

Overall, these comparisons of the replication module revealed that (a) F1 genes code for proteins that are diverse in sequence and can suffer deletions due to direct repeat sequences, (b) the F2 gene may be absent or replaced by two smaller genes, one of which (F2a) represents the first domain of F2, and (c) the F3 replicase is strongly conserved.

#### 3.3.2. ATPase (Reverse) Module

The canonical gene arrangement of R4-R5-R6-R7 is seen in the majority of plasmids (14 of 20) (Figure 4, Table 3), with four of these having an additional CDS positioned either between R5 and R4 (pBAJ9-6, pHILL-c2) or between R6 and R7 (pL6A, pISLA-s1), and in the same orientation. All (20/20) carried R5 and R6 (ATPase) genes. The six plasmids with non-canonical modules lacked homologs of one or two core genes, but the remaining genes always retained the same canonical order and orientation. Unlike the overlapping gene arrangement of the replication module, the core genes in this module often do not overlap. Genes R4 and R5 either had intergenic distances of 17–38 nt (eight cases), overlapped (seven cases), or had an intervening CDS (five cases). Genes R6 and R7 had intergenic distances between 43 and 162 nt (14/20) or had inserted genes between them (1 case). However, if atypical start codons such as CTG were utilized, then many would have potential CDS that span or nearly fill these regions. The R5/R6 genes either overlapped (5 cases) or the gene distances were small (0–3 nt distance; 14/20). No plasmid showed an inserted gene between R5 and R6. More detailed descriptions of ATPase module genes and proteins are provided in Supplementary Text S1b, which includes an analysis of accessory genes (Table S4), such as the formyltransferase encoded by pHILL-c2.

### 3.4. Relationship of pL6-Family F3 and R6 Proteins to Other Homologs

**Figure 5:**
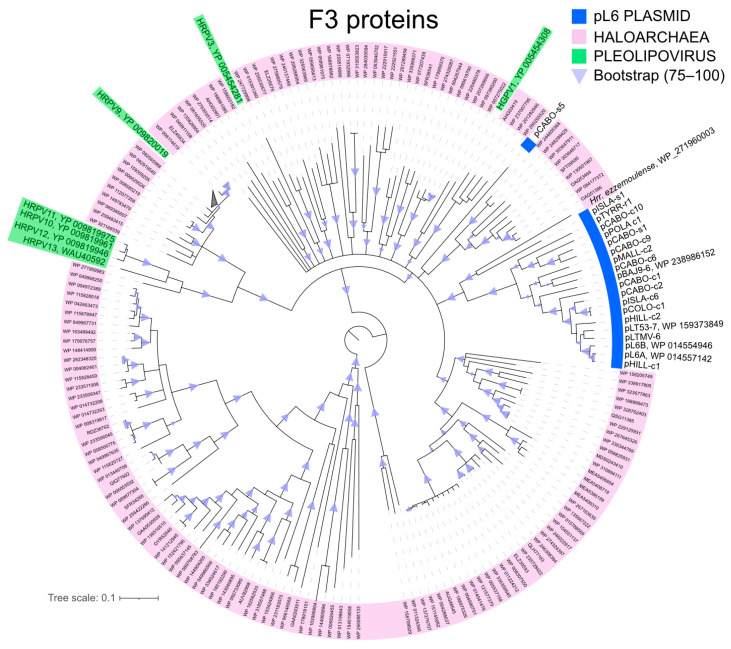
Inferred phylogeny of pL6-family F3 (replicase) proteins. Neighbor-joining consensus tree from 1000 bootstrap repetitions (see Section 2). Branches with significant bootstrap confidence values are indicated by light purple triangles. Nodes with accessions in pink shading represent proteins from members of the class *Halobacteria*. Thick blue underlining, pL6-family plasmids. Light green shading, pleolipoviruses (both accession and virus abbreviation are shown, e.g., HRPV3, Halorubrum pleomorphic virus 3).

**Figure 6:**
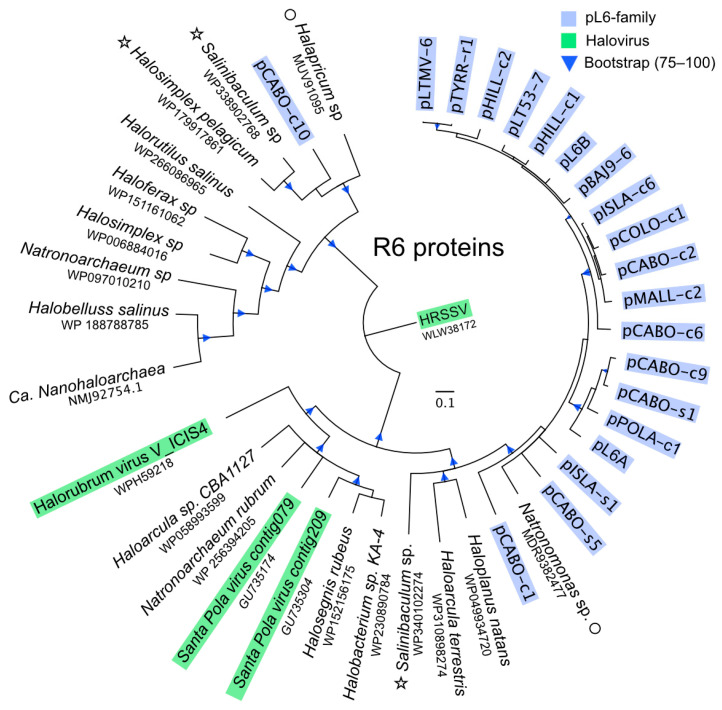
Inferred phylogeny of pL6-family R6 (ATPase) proteins. See the legend to the previous figure for details of the tree and general labeling. A circle next to the species name indicates that the protein sequence comes from a small DNA contig, and an asterisk indicates that the gene is part of a plasmid-like element (see Text).

Only the F3 (replicase) and R6 (ATPase) proteins retrieve significant numbers of closely related proteins from the sequence databases; 200 proteins for F3 but just 20 closely related proteins for R6 (BLASTp, NCBI, nr, accessed 26 April 2024, see Section 2). All matching proteins were from haloarchaea or their viruses. The phylogenetic trees inferred from these (Figure 5 and Figure 6) show that, with only a few exceptions, the pL6-family proteins cluster together and away from the proteins of known haloviruses.

In the F3 tree (Figure 5), only the proteins of the two high GC plasmids (pCABO-s5 and pISLA-s1) branch separately from the other pL6-family members. Both show long branch lengths and no close relationship to known haloviruses or to proteins with a gene neighborhood that provided any informative pattern.

In the R6 tree (Figure 6), the pCABO-c10 protein branches separately from the other pL6-family proteins and forms a clade with two cellular homologs; one from *Salinibaculum* (WP338902768) and the other from *Halosimplex* (WP179917861, HZS54_14695 in QLH82792.1). The genes for both proteins are chromosomal, and their gene contexts show that they are part of integrated elements of about 6.5 kb (Figure S9). These elements share 59.6% nt identity and are flanked at one end by a tRNA-Ala gene and at the other by an integrase gene, followed by a short direct repeat matching the end of the tRNA. They share similar gene arrangements and encoded proteins, including an alphapleolipovirus type replicase (see legend to Figure S9), but no genes for virus spike proteins. These two rare genetic elements appear to represent a group of integrative plasmids, possibly related to alphapleolipoviruses, and apart from the integrase gene, they superficially resemble the size and gene organization of pL6-family plasmids.

On the other side of the R6 tree, three of the four closest relatives of pL6-family R6 proteins are either encoded on very short contigs or their gene contexts do not provide any obvious clues, but the *Salinibaculum* protein WP340102274 is found on a 5984 bp contig with directly repeating ends that probably represents a small, circular plasmid. It carries a gene encoding a putative replicase (WP340102282, DUF1424 domain) that is unrelated to the F3 replicase of the pL6-family plasmids or haloviruses.

### 3.5. Conserved DNA Sequence Motifs

**Figure 7:**
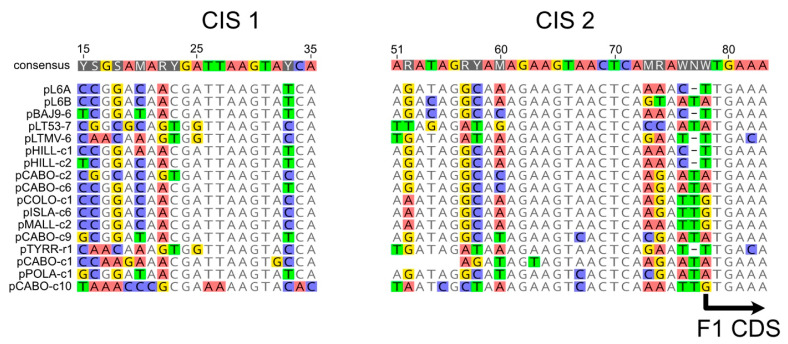
Conserved intergenic sequences (CIS) found between genes R4 and F1. Plasmid names are given on the left, and nucleotide numbers for pL6A are shown on the top. Bases are colored if they differ from the consensus at that position (threshold, 75%). The start codon for gene F1 is indicated by an arrow on the lower right.

Promoters and regulatory sequences that drive and regulate the expression of the outwardly facing gene modules of the pL6-family plasmids are probably located in the intergenic region between the R4 and F1 genes (Figure 1), upstream of both. Two conserved intergenic sequences (CIS) in this region have been described previously, one of which (CIS 1) contained a typical haloarchaeal promoter motif and the other (CIS 2) possibly being regulatory [17]. In the expanded set of twenty plasmids, the two CIS regions were conserved in 17 cases (Figure 7). The three variants not included in the alignment are pCABO-s1 and the two high GC plasmids (pCABO-s5, pISLA-s1).

### 3.6. CRISPR Spacers That Match Reconstructed pL6-Family Plasmids

**Figure 8:**
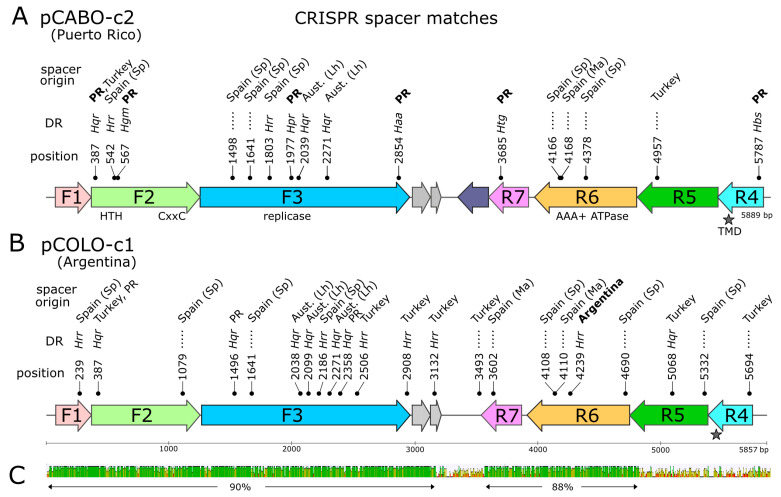
Gene map of plasmids pCABO-c2 (**A**) and pCOLO-c1 (**B**) showing the positions of the matching CRISPR spacers. For gene labeling, see the legend in Figure 1, and for spacer details, see Table S5). The position of the matching spacers are indicated above the map, with labels indicating the first matching base (e.g., 387). DR (direct repeat) level above indicates the genus (3-letter abbreviation) of the organism with the most closely related direct repeats associated with each spacer. Haa, *Halanaeroarchaeum*; Hbs, *Halobellus*; Hgm, *Halogeometricum*; Hpr, *Halapricum*; Hqr, *Haloquadratum*; Hrr, *Halorubrum*; Htg, *Haloterrigena*. The dotted lines indicate that no DR match was detected. The top level indicates the country of origin for each matching spacer: PR (Puerto Rico); Turk., Turkey; SP, Spain (Sp, Santa Pola; Ma, Mallorca); Aust. (Lh), Australia (Lake Hillier). The bold face indicates that the plasmid and spacer are from the same country. (**C**) nucleotide similarity of the two aligned plasmids (MUSCLE aligner) with regions of identity represented as green bars. Two regions of high similarity are indicated by the arrows below, along with their average nucleotide identity values.

A total of 109 spacers showing close similarity to one or more of the 15 plasmids reconstructed in this study were retrieved from the IMG/JGI CRISPR spacer database or metagenomes using the minCED tool (see Section 2) and are summarized in Table S5. All came from the metagenomes of hypersaline lakes or saltern crystallizer ponds, including the metagenomes used for plasmid reconstruction. Many matching spacers came from sites that were geographically distant from the sites from which the plasmids were derived, as depicted in Figure 8 for plasmids pCABO-c2 (Puerto Rico) and pCOLO-c1 (Argentina). Matching sequence positions are indicated above each gene map, along with the country of origin of each spacer. Matching sites are almost always located within protein-coding sequences, particularly the F3 gene encoding the replicase.

The direct repeats (DR) flanking these spacers could be used to identify the genera carrying these CRISPR arrays and, thus, the likely hosts of pL6-family plasmids (see Section 2). Over 60% of the DR (66/109) could be matched to known genera, and all of them were haloarchaea (Table S5). Half of these (33) matched the DR of *Haloquadratum* and were distributed across 10 of the 15 plasmids. In the case of pCABO-c1, all eight spacer-associated DR matched those found in *Haloquadratum*. For pHILL-c2, five out of eight DRs could be matched, and all five corresponded to the DR of *Haloquadratum*. The likely hosts for both pCABO-c1 and pHILL-c2 are members of the genus *Haloquadratum*, and this is also supported by the accessory genes they carry (Table S4). The remaining 33 DR matched a total of ten other genera of the class *Halobacteria*, most of which are commonly detected in salt lakes and crystallizer ponds, such as *Halorubrum* and *Halogeometricum* [8]. Overall, evidence from CRISPR spacers and DRs is consistent with pL6-family plasmids being present in microbial communities in hypersaline environments around the world and with their frequent invasion of haloarchaea, particularly *Haloquadratum*.

### 3.7. RNA-Seq Evidence for Transcription of pL6-Family Plasmid Genes

**Figure 9:**
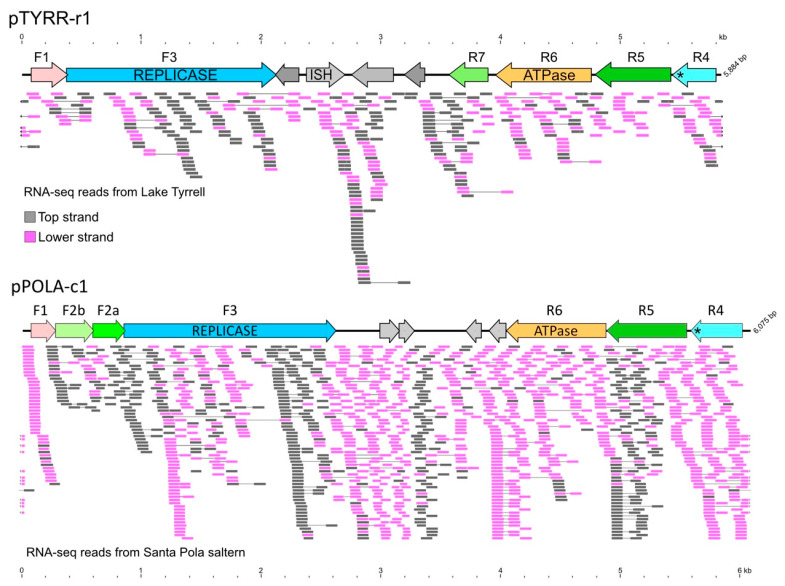
Metatranscriptome reads from Lake Tyrrell (SRR24125903) and Santa Pola Saltern (SRR10674835-40) were mapped to the sequences of plasmid pTYRR-r1 (**upper** panel) and pPOLA-c1 (**lower** panel), respectively. Mapping was performed within the Geneious Prime environment (see Section 2) with a maximum base mismatch threshold of 1%. Mapped reads are represented below each gene map and are color-coded according to strands: gray (top strand) and pink (lower strand). Lines connecting the reads indicate well-separated pairs. Arrows at the edges represent reads spanning the two edges. A scale is given above the pTYRR-r1 gene map in the upper panel and below the mapped reads in the lower panel. For clarity, most of the reads mapping to the middle of the pPOLA-c1 gene map, where the read coverage is extremely high, were removed, leaving an average coverage of 23. For gene labeling, see the legend to Figure 1.

Mapping reads from the Lake Tyrrell RNA-seq data of [10] (SRR24125903) to all pL6-family plasmids revealed that by far the most reads matched pLTMV-6 (367 reads; ≤2% mismatches), a plasmid reconstructed from metagenomic reads from the same lake. This triggered a two-step assembly of the plasmid pTYRR-r1 (see Section 2). When this plasmid was used as the reference sequence, a total of 385 matching RNA-seq reads were detected, even with a more stringent threshold of ≤1% base mismatches (Figure 9, upper panel). All but 38 nt of the plasmid were represented by RNA-seq reads, with a distinct peak of read coverage (24.5×) across the gene for an insertion sequence (ISH) and an adjacent CDS (nt 2379–3107). Since the RNA-seq data of Le Lay et al. are strand-specific (Illumina TruSeq), the reads have been color-coded in the figure to indicate the strand (gray, top strand; pink, lower strand).

Metagenomic RNA-seq data are also available for the Santa Pola saltern (SRR10674835-40), and these were mapped to pPOLA-c1, a plasmid reconstructed in the present study from the metagenome of the same saltern (Figure 9, lower panel). A total of 78,582 paired reads were mapped to this plasmid (stringency of ≤1% mismatches), but most reads matched to two adjacent, small CDS located near the middle of the plasmid (280 bp; nt 2992–3271) in a region where insertions of foreign genes are often found in other pL6-family plasmids [17]. These two small CDS are similar to chromosomal sequences of *Hqr. walsbyi* that often occur near HqIRS55 insertion sequences (e.g., FR746099.1, nt 1,362,480–1,362,865). Ignoring the central, high-coverage region, reads matching the upper and lower strands were common throughout the entire sequence of pPOLA-c1.

The presence of reads mapping to both strands across most of the annotated genes of both plasmids indicates not only active transcription of their genes, but also widespread counter-transcription. High levels of counter-transcription have been observed in haloviruses such as HF2 [37] and SH1 [38].

## 4. Discussion

Fifteen pL6-family plasmids were reconstructed in this study from the metagenomes of salt lakes and saltern crystallizer pools in four widely separated countries: Argentina, Australia, Puerto Rico, and Spain. This increases the number of complete plasmid sequences from five to twenty and greatly expands their geographical distribution. All share the archetypical gene arrangement of outward-facing replication and ATPase modules reported previously [17], and while nearly half of the plasmids (7 of 15) displayed a closely similar gene complement to the five earlier examples, some novel variants were discovered, such as those where the F2 gene has been replaced with two smaller genes (F2b/F2a) or lost entirely. The significance of these differences remains unresolved, as the molecular functions of the proteins encoded on pL6-family plasmids are largely unknown and their sequences provide few clues.

The association of pL6-family plasmids with *Hqr. walsbyi* seen previously [17], is supported by the findings of the current metagenomic study. CRISPR DR sequences indicated that at least two plasmids (pCABO-c1, pHILL-c2) are from this species. This is also consistent with the accessory (acquired) genes of these two plasmids, which have encoded proteins most similar to homologs from *Hqr. walsbyi*. The high community abundance of *Haloquadratum* in Puerto Rico (53.8 to 69.8%), Lake Tyrrell (58%), and Santa Pola (63%) saltern ponds [1,8] also makes it likely that plasmids recovered from these sites are harbored by this species, which would include the pCABO-series (except pCABO-s5, see below), pTYRR-r1, and pPOLA-c1. In the cases of pCABO-s1 and pTYRR-r1, their accessory genes also support *Hqr. walsbyi* as being the host. Although DR sequences from genera other than *Haloquadratum* were observed, suggesting that pL6-family plasmids can spread to other species, no examples have yet been reported in the literature, nor can pL6-family plasmids be found in the currently available (July 2024) complete genome sequences of cultivated haloarchaea. Why these plasmids appear to be most commonly carried by *Hqr. walsbyi* remains to be unraveled.

The avoidance of specific palindromic motifs is common in mobile genetic elements and viruses of prokaryotes in order to circumvent restriction-modification (RM) defense systems of host cells [39,40]. Many RM systems of haloarchaea have been documented [41,42]. In the current study, tetramer analysis confirmed the general avoidance of GGCC and CTAG motifs in pL6-family plasmids, as reported previously [4]; however, in this larger dataset, there were two prominent exceptions. The two high GC plasmids, pCABO-s5 and pISLA-s1, maintain both motifs at the expected frequency. Because the sequenced strains of *Hqr. walsbyi* (C23T and HBSQ001) avoid GGCC and CTAG motifs [4], and carry multiple restriction systems, one of which is predicted to recognize GGCC motifs (Hqrw_2139; REBASE, http://rebase.neb.com, accessed June 10, 2024). The atypical sequences of pCABO-s5 and pISLA-s1 suggest that their usual hosts are haloarchaeal species other than *Haloquadratum*.

The case of pHILL-c2, where GGCC is absent and CTAG sites are not under-represented, is best explained by the acquisition of foreign DNA by this plasmid that contains multiple CTAG sites. The acquired DNA carries an accessory gene encoding a formyltransferase, and using the predicted 3D structure of this enzyme, it was identified as a sugar N-formyltransferase that probably synthesizes dTDP-4-formamido-4,6-dideoxyglucose. In bacteria like *Mycobacterium tuberculosis*, the N-formylated sugar ends up in cell surface polysaccharides [43,44]. Further evidence for its natural function was sought by examining the gene neighborhoods for closely matching proteins, which showed that adjacent genes commonly encoded glycosyltransferases or enzymes involved in polysaccharide biosynthesis, supporting the function obtained from the 3D structure similarity results. Two other accessory genes carried by the pL6-family plasmids were examined in the same way. Both encode methyltransferases, and the genes surrounding the most closely matching proteins are frequently glycosyltransferases. Taken together, these results suggest that enzymes involved in sugar modification or addition provide a selective advantage for pL6-family plasmids, possibly involving the alteration of glycans attached to cell surface proteins or cell surface polysaccharides.

The possibility that pL6-family plasmids represent a novel group of temperate viruses was suggested by the studies of Lake Tyrrell, where water filtered down to 0.1 µm was used to produce ‘virus concentrates’ [45], the DNA of which had abundant reads to this plasmid family and were used to reconstruct plasmid pLTMV-6. However, filtration not only recovers virus particles but also dissolved DNA released from lysed cells, extracellular membrane vesicles (EV) [46], and membrane-enveloped plasmids [47,48]. Viruses are normally purified using a combination of methods, including density gradient centrifugation and ultracentrifugation. In a recent study [11], Santa Pola crystallizer water was fractionated into dissolved DNA (by filtration) and virus pellets (by ultracentrifugation). DNA sequence reads from those fractions were used in the present study to determine the relative abundance of plasmid pPOLA-c1 in these fractions. Compared to cellular DNA, pPOLA-c1 reads were 1.6–2.6× higher in dissolved DNA fractions but 34-fold lower in the virus pellet fraction. An unexplained over-representation of plasmids in the dissolved DNA fractions was also noted by Aldeguer-Riquelme et al., who found a two-fold higher abundance of *Hqr. walsbyi* plasmid pL47 in dissolved compared to intracellular DNA [11], and they speculated that this might be due to plasmid resistance to exonucleases or by export from cells in membrane vesicles [48]. In any case, the very low level of pPOLA-c1 in the virus pellet is evidence against the formation of virus particles that could be sedimented by ultracentrifugation. Three possible interpretations of these data are that (a) pPOLA-c1 exists only as a plasmid, (b) pPOLA-c1 is a temperate virus, but induction rates are extremely low, and (c) the fractionation methods used by [11] did not pellet low-density or delicate (e.g., lipid enveloped) viruses. Indeed, their analysis identified only tailed viruses (Class *Caudoviricetes*) in the virus pellets.

Transcription of pL6-family plasmids has not been reported previously and was only possible because of publicly available strand-specific metatranscriptome data from Lake Tyrrell and Santa Pola, which could be interrogated using plasmids reconstructed from metagenomes obtained from the same sites. These results provide unexpected insights into plasmid gene expression. Hundreds of closely matching paired reads matched sequences on both strands and across all genes of pTYRR-r1 and pPOLA-c1. The matching transcriptome reads provide independent support for the reconstructed plasmid sequence of pPOLA-c1 and for most of pTYRR-r1. While there are many unknown variables in these results, they imply (a) significant levels of these plasmids were present in the corresponding microbial communities, sufficient for the detection of their transcripts, and (b) that widespread transcription occurs from both DNA strands, producing both sense and antisense transcripts. Antisense or counter-transcripts have commonly been observed in transcriptome studies of prokaryotes [49], including haloarchaea [50,51,52,53], and are generally thought to be involved in the regulation of gene expression [50]. A study on *Halobacterium salinarum* found that at least 21% of the genes produced antisense transcripts, but most were at low levels [52]. In the haloviruses that have been studied, antisense transcripts are commonly detected [38].

Although additional examples of pL6-family plasmids recovered in this study have shed light on their conserved and variable features, the underlying biology of these plasmids remains poorly understood. CRISPR spacer matches show that they invade haloarchaeal cells, but how are they transferred or gain entry, and what genes are involved in maintenance or partition? Are they merely plasmids, defective remnants of temperate pleolipoviruses, or perhaps a novel type of virus? Their sequences show features similar to those of lipid-enveloped pleolipoviruses [19,54,55], particularly the betapleolipovirus group, which has a similar replicase gene and, like many other viruses, displays strong conservation of gene arrangement with colinear, often overlapping, genes that form functional modules. Such arrangements allow viruses to rapidly and efficiently produce virions during productive infection while maintaining genome sizes within packaging limits. In contrast to viruses, plasmids have far fewer constraints and are more likely to vary in size and gene complement [56,57]. While pL6-family plasmids maintain a conserved gene arrangement and limited size, they do not encode a membrane-anchored protein related to the spike proteins of pleolipoviruses (or any other virus). However, the capsid proteins of viruses are generally well-conserved in structure, permitting relationships to be discerned across wide evolutionary distances [58]. The only predicted membrane-anchored protein encoded by the pL6-family plasmids is R4. Finally, ultracentrifugation of filtered saltern water from Santa Pola [11] showed no evidence that the pL6-family plasmids are packaged within capsids or lipid envelopes. Our analysis provides a foundation for future studies to elucidate the true nature of these intriguing genetic elements, the functions of their proteins, and how they regulate gene expression, including the role of antisense transcripts.

## Figures and Tables

**Table 1:** The fifteen pL6-family plasmids reconstructed in this study, along with the five previously reported plasmids (gray shaded) ^a^.

Country	Plasmid Name ^b^	Size (bp)	%G+C	Source	Metagenome BioProject ^c^	Plasmid Accession
Argentina	pCOLO-c1	5857	53.9	Laguna Colorada Chica	PRJEB45291	BK067852
Australia	pL6A	6129	51.0	*Hqr. walsbyi* C23	NA ^d^	FR746101
Australia	pL6B	6056	52.0	*Hqr. walsbyi* C23	NA	FR746102
Australia	pBAJ9-6	6213	53.0	*Hqr. walsbyi* Bajool9	NA	LT984491
Australia	pLT53-7	7045	51.0	*Hqr. walsbyi* LT53-19	NA	LT984489
Australia	pLTMV-6	5884	53.0	Lake Tyrrell	PRJNA81851	LT991975
Australia	pHILL-c1	6187	52.4	Lake Hillier	PRJNA865792	BK067853
Australia	pHILL-c2	7625	51.5	Lake Hillier	PRJNA865792	BK067854
Australia	pTYRR-r1	5844	51.0	Lake Tyrrell	PRJNA81851	BK067859
Puerto Rico	pCABO-c1	5007	48.4	Cabo Rojo Saltern	PRJNA343765	BK067804
Puerto Rico	pCABO-c2	5889	53.7	Cabo Rojo Saltern	PRJNA343765	BK067846
Puerto Rico	pCABO-c6	5864	53.0	Cabo Rojo Saltern	PRJNA343765	BK067847
Puerto Rico	pCABO-c9	6930	49.3	Cabo Rojo Saltern	PRJNA343765	BK067848
Puerto Rico	pCABO-c10	5219	51.4	Cabo Rojo Saltern	PRJNA343765	BK067849
Puerto Rico	pCABO-s1	6206	47.8	Cabo Rojo Saltern	PRJNA343765	BK067850
Puerto Rico	**pCABO-s5**	5017	**58.5**	Cabo Rojo Saltern	PRJNA343765	BK067851
Spain (Isla Cristina)	pISLA-c6	5881	53.6	Isla Cristina Saltern	PRJNA890281	BK067855
Spain (Isla Cristina)	**pISLA-s1**	5606	**64.0**	Isla Cristina Saltern	PRJNA890281	BK067856
Spain (Mallorca)	pMALL-c2	5504	52.6	s’Avall Saltern	PRJEB45291	BK067857
Spain (Santa Pola)	pPOLA-c1	6075	48.1	Santa Pola Saltern	PRJNA713327	BK067858

^a^ Gray shaded rows are the five plasmids reported previously [17], mostly from strains of *H. walsbyi*. These are included to compare with the plasmids reconstructed in the present study. Bold type (pCABO-s5, pISLA-s1, and their %G+C values) indicate the two ‘high GC’ plasmids. ^b^ Except for previously reported plasmids (gray shaded), plasmid names indicate the source and assembly method. The suffix c is used for de novo assembled contig sequences, which are extended by repeated rounds of read mapping. The suffix s denotes that the assembly used metaplasmidSpades (see Section 2). The r suffix indicates that this plasmid was extended by read mapping from a contig derived from RNA-seq data. ^c^ NCBI Bioproject accession number of the metagenome read data used to reconstruct each plasmid. ^d^ NA, not applicable, as these were sequenced from isolates not reconstructed from metagenomes.

**Table 2:** Relative levels of pPOLA-c1 in filtered and centrifuged fractions of Santa Pola water ^a^.

DNA Fraction	Fraction Name ^b^	Fraction Description ^b^ (Accession)	# Mapped Reads to pPOLA-c1	Relative Proportion of Mapped Reads
Cell pellet	iDNA	intracellular DNA (SRR13926770)	1228	1
Supernatant after ultracentrigation	FdDNA	protocol F, dissolved DNA (SRR13926769)	3217	2.6
Supernatant after ultracentrigation	CdDNA	protocol C, dissolved DNA (SRR13926768)	1982	1.6
Pellet from ultracentrifugation	vDNA	viral DNA (SRR13926767)	39	0.03

^a^ Metagenomic reads from the study of [11] were mapped to the sequence of plasmid pPOLA-c1 (BK067858) using the ‘map to reference’ tool within Geneious Prime v. 2024.0.5 (Geneious mapper, ≤1% base mismatches). Three million paired reads were used from each of the four fractions (accessions given in the Fraction Description column). ^b^ Fraction names and Fraction Descriptions are from [11].

**Table 3:** Summary of gene variation across pL6-family plasmids ^a^.

		Core Genes						
Plasmid	Variant	F1	F2	F3	R4	R5	R6	R7
pL6A	canonical	F1	F2	F3	R4	R5	R6	R7
pL6B	canonical	F1	F2	F3	R4	R5	R6	R7
pLTMV-6	canonical	F1	F2	F3	R4	R5	R6	R7
pLT53-7	canonical	F1	F2	F3	R4	R5	R6/ps.	R7
pBAJ9-6	canonical	F1	F2	F3	R4	R5	R6	R7
pCABO-c6	canonical	F1	F2	F3	R4	R5	R6	R7
pCABO-c2	canonical	F1	F2	F3	R4	R5	R6	R7
pCABO-c9	canonical	F1	F2	F3	R4	R5	R6	-
pHILL-c2	canonical	F1	F2	F3	R4	R5	R6	R7
pCOLO-c2	canonical	F1	F2	F3	R4	R5	R6	R7
pISLA-c6	canonical	F1	F2	F3	R4	R5	R6	R7
pHILL-c1	canonical	F1	F2	F3	R4	R5	R6	R7
pTYRR-r1	no_F2	F1	-	F3	R4	R5	R6	R7
pCABO-c1	no_F2	F1	-	F3	R4	R5	R6	-
pCABO-c10	no_F2	F1	-	F3	R4	R5	R6	-
pMALL-c2	F2b/F2a	F1	F2b/F2a	F3	R4	R5	R6	R7
pPOLA-c1	F2b/F2a	F1	F2b/F2a	F3	R4	R5	R6	-
pCABO-s1	F2b/F2a	F1	F2b/F2a	F3	-	R5	R6	-
pISLA-s1	F1b/F2a/F2c	F1b	F2a/F2c	F3	R4	R5	R6	R7
pCABO-s5	F1b/F2a/F2c	F1b	F2a/F2c	F3	R4	R5	R6	-

^a^ See Figure 4 and Supplementary Text S1 for details of the variant names and core genes. The gray shading of the first five plasmids denotes those previously described. Accessory genes are not shown but are listed in Table S4. In column R6, ps. indicates pseudogene for plasmid pLT53-7. The absence of a core gene is indicated by “-”.

## Data Availability

The 15 plasmid sequences described in this study are available from the GenBank database with the following accessions: BK067804, BK067846–BK067859.

## Supplementary Materials

The following supporting information can be downloaded at: https://www.mdpi.com/article/10.3390/genes15091123/s1, Figure S1: (panels A to O): Read mapping to reconstructed pL6-family plasmids; Figure S2: (panels A to I): Alignments of pL6-family plasmid proteins; Figure S3: Structural comparison of F2 and F2a proteins; Figure S4: AlphaFold2 prediction of F2b structure (pPOLA-c1); Figure S5: AlphaFold2 structure prediction of halovirus His1_gp16 (AAQ13731); Figure S6: Comparison of R6 proteins from pL6A and pCABO-c10; Figure S7: Close structural similarity between formyltransferase encoded by pHILL-c2 and *M. tuberculosis* dTDP-4-amino-4,6-dideoxyglucose formyltransferase (P9WKZ3); Figure S8: A: Gene neighbors *of T. thiocyanaticus* SAM-dependent methyltransferase WP_125180990; B: Gene neighbors of *Har. marina* DT1 FkbM family methyltransferase WP_254272245.1; C: Gene neighbors of *Hbt. salinarum* UC4238 gene encoding formyltransferase MDL0133728 (WP_285266011); Figure S9: Comparison of plasmids pLTMV-6 and pTYRR-r1; Figure S10: Comparison of two integrative elements containing R6-homologs; Table S1: Metagenome, metavirome and RNA-seq read data used in this study [59,60,61]; Table S2: Mapping of reads to reconstructed pL6-like plasmids; Table S3: Absent or under-represented tetramers in pL6-plasmids; Table S4: Accessory genes of pL6-family plasmids and their top database matches; Table S5: CRISPR spacers matching pL6-family plasmids reconstructed in this study; Text S1: a: Detailed descriptions of pL6-family plasmid replication modules; b: Detailed descriptions of pL6-family plasmid ATPase modules.
