# Supplementary material for: Global Distribution and Diversity of Haloarchaeal pL6-Family Plasmids
Source: Genes (Basel). 2024 Aug 26;15(9):1123. doi: 10.3390/genes15091123 (PMC11431627; doi:10.3390/genes15091123)
Supplement: Supplementary file 1 [file genes-15-01123-s001.zip › Supplementary text S1.pdf]

Supplementary text S1a. Detailed descriptions of pL6-family plasmid replication modules

An alignment of F1 proteins (Figure S2A) shows considerable sequence diversity ranging from (a) identical or near-identical sequences (pCOLO-c1, pISLA-c6, pMALL-c2), (b) sequences showing deletions (e.g. pCABO-c6), and (c) the highly divergent F1b sequences of pISLA-s1, pCABO-s5. None contain conserved protein domains or match proteins with known function. Alphafold2 did not predict any tertiary structure. Those proteins with deletions were seen to have suffered losses within the c-terminal half, after approximately residue 50. Examination of full-length F1 gene sequences revealed the presence of direct repeats, 16-27 nt in length, that occur within the distal half, from nt 199 onwards. For example, pCOLO-c1 carries three 17 nt direct repeats of TTGCACCAACTCGTGCA between nt 277-353. These nucleotide repeats are a likely cause for deletion events within these genes.

F2 proteins have few matches in the NCBI databases (BLASTp, nr database, accessed April 22, 2024) and do not carry conserved domains that could indicate function. The twenty plasmids encode proteins that have been divided into four groups. Groups F2 and F2a represent the majority (17) and are related by sequence and structure. The F2 proteins from the twelve plasmids with canonical gene arrangement have similar lengths (296-301 aa), sequence (71-100% aa identity), and all possess a CxxC motif near the C-terminus (Figure S2B). Their alphafold2 predicted structures show two compact domains that are connected by a flexible linker (e.g. Figure S3). The five F2a proteins are much shorter (87 aa), share 31-100% aa identity to each other, and align to the N-terminal end of the canonical F2 proteins (32-50% aa identity). Their sequences encompass the first F2 domain, and alphafold2 predictions show F2a proteins fold with high confidence to form structures very similar to that of the domain 1 of F2 (Figure S3). The F2a genes of three plasmids are positioned immediately upstream of F3 while in two plasmids this gene is separated from F3 by a short, unrelated CDS (F2c, see below).

In the third group, three plasmids have an F2b gene positioned between F1 and F2a. An alignment is given in Figure S2C. The three F2b proteins are of similar length (105-109 aa) and closely related (77-97% aa identity) but show no sequence similarity to other pL6 proteins, including F2, no detectable conserved domains and no matches to proteins in GenBank (BLASTp, nr database, accessed April 22, 2024). Structural predictions using alphafold2 showed they could form a compact domain consisting of three short alpha helices and two beta strands (Figure S4) but much of this structure was of low confidence, it did not match domain 2 of F2, and structure similarity searches (https://search.foldseek.com/) did not return any significant matches.

The last group are the two F2c proteins found only in the two high GC plasmids. They are relatively short (52 and 72 aa) and share 20% aa identity (Figure S2D). The F2c of pISLA-s1 has two CxxC motifs. Neither protein retrieved matches from NCBI using BLASTp (nr database, accessed April 22, 2024).

F3 proteins are predicted to represent a novel replicase [1,2], and their alignment is shown in Figure S2E. Except for the proteins from the two high-GC plasmids (pCABO-s5, pISLA-s1) the other 18 share high sequence similarity (62-99% aa identity). The 99% aa identity of F3 proteins from pISLA-c6 and pCOLO-c1 reflects the almost identical nucleotide sequence of the replication modules of these two plasmids (99.6% nt similarity; Figure 4).

When the twenty F3 protein sequences are used to search against virus proteins (BLASTp, NCBI nr, viruses (taxid:10239), accessed 22 April 2024), the top two matches in all cases are the putative replicases of the betapleolipoviruses Halorubrum pleomorphic virus 3 (HRPV3, HRPV-3_gp09, YP_005454281) and Halogeometricum pleomorphic virus 1 (HGPV1, HGPV-1_gp14, YP_005454308), with 42-50% aa identity.

Overall, these comparisons of the replication module revealed that (a) F1 genes code for proteins that are diverse in sequence and can suffer deletions due to direct repeat sequences, (b) the F2 gene may be absent or be replaced by two smaller genes, one of which (F2a) represents the first domain F2, and (c) the F3 replicase is strongly conserved.

Supplementary text S1b. Detailed descriptions of pL6-family plasmid ATPase modules.

R4 genes. All but one plasmid carried a R4-family gene. The encoded proteins range in length from 90-134 aa and vary widely in sequence (5 - 100% aa identity; Figure S2F) but all carry transmembrane domains at their C-termini, indicated by black asterisks in Figure 4. BLASTp searches of NCBI return only pL6-family homologs (accessed 22 April 2024). The single plasmid without an R4 gene (pCABO-s1) also has an unusual ATPase module that includes a foreign gene (encoding a HTH domain protein) located between genes R6 and (putative) R7, but in the opposite orientation (see below).

R5 genes. The predicted R5 proteins varied widely in sequence (12 - 98% aa identity) but all possessed a serine as the second amino acid and all except one had the C-terminal sequence (F/Y)DYSDLI (Figure S2G). BLASTp searches of NCBI (accessed 22 April 2024) returned very few matches, with almost all being previously published pL6-family homologs. The most divergent R5 protein is that of pCABO-c10, which also lacks the C-terminal motif. The alphafold2 predicted structures of R5 proteins, such as pL6A R5 (https://www.alphafold.ebi.ac.uk/entry/G0LNF8), show a compact central domain with three alpha-helices while the C-terminal conserved motif lies at the end of a long flexible tail. The extreme conservation of the C-terminal motif across widely divergent R5 proteins indicates an important function.

R6 (ATPase protein) genes. All have predicted P-loop NTPase domains and C-terminal HTH domains and they share 60-99% aa identity except for the R6 protein of pCABO-c10 (Figure S2H). The pCABO-c10 protein is only distantly related to the others (19-25% aa identity) but alphafold2 predictions show it has a closely similar structure (Figure S6), with a compact P-loop ATPase domain and a helix-turn-helix domain, connected by a flexible linker. A number of similar proteins can be retrieved from the protein databases using BLASTp searches (NCBI, nr proteins) and their diversity is described further below.

R6 proteins show up to 24% aa identity with spindle-shaped halovirus His1 protein His1V_gp16 (AAQ13731), an ATPase [3,4]. An alphaFold2 structural prediction was performed to test whether their 3D structures were also similar. As shown in Figure S5 the structure of His1V_gp16 is closely similar to the R6 proteins of pL6B and pCABO-c10 (Figure S6), with the same overall architecture. Two recently discovered haloviruses also carry R6 homologs. Halorubrum spindle-shaped virus-BLv25 (OQ850971, HRSSV) encodes a protein (WLW38172, named ORF1) sharing 26% aa identity with R6 of pL6B, and Halorubrum virus V_ICIS4 (OR762182.1) specifies a protein (WPH59218, AFNJKBDN_CDS0001) that is 39% identical to pL6B R6.

R7 genes. Most plasmids (14/20) carried R7 genes, and their encoded proteins are closely related (61-97% aa identity; Figure S2I). BLASTp searches (NCBI, nr, accessed 22 April 2024) retrieve only the previously reported pL6-family homologs. Predicted R7 protein structures (e.g. pL6A, AF-G0LNF6-F1) show a compact arrangement of central beta-strands surrounded by three alpha-helices. Among the other six plasmids that lack an R7 gene, two carry genes in a similar position and orientation. The first is pCABO-s1 (nt 3,403-3,032) which encodes a protein with a terminal phenylalanine (as do all R7 proteins), but its overall protein similarity to R7 proteins is insignificant. The second is pCABO-c1 (nt 2,890-2,489), which instead encodes an unrelated protein matching *Haloquadratum* proteins such as Hqrw_2237.

Accessory genes. Some plasmids carry one or two genes that are unrelated to the core genes and appear to have been acquired by insertion into the plasmid backbone (Figure 4, grey coloring). Most are found in the region between the ends of genes F3 and R7, downstream in both cases, presumably because insertions there have little impact on the functions of replication and ATPase gene modules. Eight accessory genes (seven encoding proteins and one insertion sequence) are listed in Table S4, along with their top database matches. In all but one case, the top match was from haloarchaea, and the most common genus was *Haloquadratum*. Three proteins have general functional assignments: two methyltransferases and a formyltransferase but their natural substrates are not known.

Structural similarity of the formyltransferase enzyme specified by pHILL-c2 supported a role in sugar modification. The alphafold2 predicted structure shares close resemblance to sugar N-formyltransferases, such as dTDP-4-amino-4,6-dideoxyglucose formyltransferase from *Mycobacterium tuberculosis* (AF-P9WKZ3-F1-model_v4; foldseek E-value, 9.0 x 10^{-22}; https://search.foldseek.com, accessed July 20, 2024). This enzyme converts dTDP-4-amino-4,6-dideoxyglucose into dTDP-4-formamido-4,6-dideoxyglucose and its structure has been experimentally determined [5,6]. A structural alignment with the pHILL-c2 protein is provided in Figure S7.

The gene contexts of the corresponding best matching proteins of the three transferases were examined (Figure S8, A-C). All are nearby to genes encoding enzymes involved in sugar transfer, synthesis or modification (e.g. glycosyltransferases, sialic acid synthase, polysaccharide biosynthesis protein SpsG).

References

1. Krupovic, M.; Cvirkaite-Krupovic, V.; Iranzo, J.; Prangishvili, D.; Koonin, E.V. Viruses of archaea: structural, functional, environmental and evolutionary genomics. *Virus Res.* **2017**, *244*, 181-193.
2. Alarcon-Schumacher, T.; Lucking, D.; Erdmann, S. Revisiting evolutionary trajectories and the organization of the *Pleolipoviridae* family. *PLoS Genet.* **2023**, *19*, e1010998.
3. Dyall-Smith, M.L.; Pfeiffer, F.; Klee, K.; Palm, P.; Gross, K.; Schuster, S.C.; Rampp, M.; Oesterhelt, D. *Haloquadratum walsbyi*: limited diversity in a global pond. *PLoS One* **2011**, *6*, e20968.
4. Dyall-Smith, M.; Pfeiffer, F. The PL6-family plasmids of *Haloquadratum* are virus-related. *Front. Microbiol.* **2018**, *9*, 1070.
5. Girardi, N.M.; Thoden, J.B.; Holden, H.M. Misannotations of the genes encoding sugar N-formyltransferases. *Protein Sci.* **2020**, *29*, 930-940.
6. Dunsirn, M.M.; Thoden, J.B.; Gilbert, M.; Holden, H.M. Biochemical investigation of Rv3404c from *Mycobacterium tuberculosis*. *Biochemistry* **2017**, *56*, 3818-3825.
