# Supplementary material for: Global Distribution and Diversity of Haloarchaeal pL6-Family Plasmids
Source: Genes (Basel). 2024 Aug 26;15(9):1123. doi: 10.3390/genes15091123 (PMC11431627; doi:10.3390/genes15091123)
Supplement: Supplementary file 1 [file genes-15-01123-s001.zip › Table_S1_reads_v33.pdf]

Supplementary Table S1. Metagenome, metavirome and RNA-seq read data used in this study

Country	Site	Year[a]	Reads[b]	Accession[c] (sample/type)	Reference[d]
Argentina	Salina Laguna Colorada Chica	2019	2 × 150 nt	ERR7916263	(Viver *et al.*, 2023)
Australia	Lake Hillier	2015	2 × 250 nt	SRR26978067 (FW-E3) SRR26968577 (FW-D3)	(Sierra *et al.*, 2022)
Australia	Lake Tyrrell	2009 2009 2010 2010	Average = 515 nt	SRR5637210 SRR5637211 SRR402042 SRR402044 (virus concentrates)	(Podell *et al.*, 2014)
Australia	Lake Tyrrell	2018	2 × 100 nt	SRR24125903-4 (RNA-seq)	(Le Lay *et al.*, 2023)
Puerto Rico	Cabo Rojo Saltern	2014	2 × 250 nt	SRR8816317 (sample 1)	(Couto-Rodriguez and Montalvo-Rodriguez, 2019)
Puerto Rico	Cabo Rojo Saltern	2016	2 × 150 nt	SRR8816318 (sample 2) SRR8816319 (sample 3)	(Couto-Rodriguez and Montalvo-Rodriguez, 2019)
Spain	Isla Cristina saltern	2020	2 × 150 nt	SRR23092357 SRR21894959	(Garcia-Roldan *et al.*, 2023)
Spain	Alicante, Santa Pola saltern, CR30	2019	2 × 250 nt	SRR13926770	(Aldeguer-Riquelme *et al.*, 2021)
Spain	Mallorca, s'Avall solar saltern	2018	2 × 150 nt	ERR5979340	(Viver *et al.*, 2023)
Spain	Alicante, Santa Pola brines	2019	2 × 100 nt	SRR10674835-40 (RNA-seq)	PRJNA595057 Reg. date: 12-Dec-2019 Centro de Astrobiologia

[a] Year of sampling or year of release of sequence data if sampling date is not given.
[b] Most reads are paired (2 ×). Read lengths given in nucleotides (nt). RNA-seq reads are strand-specific.
[c]Accessions from ENA (https://www.ebi.ac.uk/ena/browser/home) or SRA (https://www.ncbi.nlm.nih.gov/sra).
[d]Either a publication or an NCBI BioProject ID (https://www.ncbi.nlm.nih.gov/bioproject).

References

Aldeguer-Riquelme, B., Ramos-Barbero, M.D., Santos, F., and Anton, J. (2021). Environmental dissolved DNA harbours meaningful biological information on microbial community structure. *Environ Microbiol* 23(5), 2669-2682. doi: 10.1111/1462-2920.15510.

Couto-Rodriguez, R.L., and Montalvo-Rodriguez, R. (2019). Temporal analysis of the microbial community from the crystallizer ponds in Cabo Rojo, Puerto Rico, using metagenomics. *Genes (Basel)* 10(6). doi: 10.3390/genes10060422.

Garcia-Roldan, A., de la Haba, R.R., Vera-Gargallo, B., Sanchez-Porro, C., and Ventosa, A. (2023). Metagenomes of a crystallizer pond from Isla Cristina saltern in Spain. *Microbiol Resour Announc* 12(5), e0003923. doi: 10.1128/mra.00039-23.

Le Lay, C., Hamm, J.N., Williams, T.J., Shi, M., Cavicchioli, R., and Holmes, E.C. (2023). Viral community composition of hypersaline lakes. *Virus Evolution* 9(2), 1-14. doi: 10.1093/ve/vead057.

Podell, S., Emerson, J.B., Jones, C.M., Ugalde, J.A., Welch, S., Heidelberg, K.B., et al. (2014). Seasonal fluctuations in ionic concentrations drive microbial succession in a hypersaline lake community. *ISME J* 8(5), 979-990. doi: 10.1038/ismej.2013.221.

Sierra, M.A., Ryon, K.A., Tierney, B.T., Foox, J., Bhattacharya, C., Afshin, E., et al. (2022). Microbiome and metagenomic analysis of Lake Hillier Australia reveals pigment-rich polyextremophiles and wide-ranging metabolic adaptations. *Environ Microbiome* 17(1), 60. doi: 10.1186/s40793-022-00455-9.

Viver, T., Conrad, R.E., Lucio, M., Harir, M., Urdiain, M., Gago, J.F., et al. (2023). Description of two cultivated and two uncultivated new *Salinibacter* species, one named following the rules of the bacteriological code: *Salinibacter grassmerensis* sp. nov.; and three named following the rules of the SeqCode: *Salinibacter pepae* sp. nov., *Salinibacter abyssi* sp. nov., and *Salinibacter pampae* sp. nov. *Syst Appl Microbiol* 46(3), 126416. doi: 10.1016/j.syapm.2023.126416.
