# Supplementary material for: Global Distribution and Diversity of Haloarchaeal pL6-Family Plasmids
Source: Genes (Basel). 2024 Aug 26;15(9):1123. doi: 10.3390/genes15091123 (PMC11431627; doi:10.3390/genes15091123)
Supplement: Supplementary file 1 [file genes-15-01123-s001.zip › Table_S2_coverage_v33.pdf]

## Supplementary Table S2. Mapping of reads to reconstructed pL6-like plasmids.

| Plasmid[a] | Size (bp) | Reads[b] | Reads mapped[c] (threshold %) | Coverage (Fold) | Total Reads | Similarity (%)[d] |
|---|---|---|---|---|---|---|
| pCOLO-c1 | 5,857 | ERR7916263 | 1,744 (0%) | 42 | 72,140,396 | 100 |
| pHILL-c1 | 6,187 | SRR26978067 + SRR26968577 | 4,213 (1%) | 147 | 56,053,528 + 17,295,462 | 99.7 |
| pHILL-c2 | 7,625 | SRR26978067 | 580 (1%) | 18 | 17,295,462 | 99.8 |
| pTYRR-r1 | 5,844 | SRR5637210 | 391 (1%) | 28 | 1,052,913 | 99.3 |
| pCABO-c1 | 5,007 | SRR8816317 | 1,504 (0%)<br>----------------<br>2,876 (1%) | 74<br>-----------<br>142 | 28,870,606<br>------------<br>28,870,606 | 100<br>--------<br>99.7 |
| pCABO-c2 | 5,889 | SRR8816317 | 1122 (1%) | 47 | 28,870,606 | 99.7 |
| pCABO-c6 | 5,864 | SRR8816317 | 1177 (1%) | 50 | 28,870,606 | 99.7 |
| pCABO-c9 | 6,930 | SRR8816317 | 556 (1%) | 20 | 28,870,606 | 99.7 |
| pCABO-c10 | 5,219 | SRR8816317 | 772 (1%) | 37 | 28,870,606 | 99.7 |
| pCABO-s1 | 6,206 | SRR8816317 | 998 (1%) | 40 | 28,870,606 | 99.7 |
| pCABO-s5 | 5,017 | SRR8816317 + SRR8816318 | 1,053 (1%) | 35 | 28,870,606 25,000,000 | 99.9 |
| pISLA-c6 | 5,881 | SRR21894959 SRR23092357 | 1,327 (1%) | 33 | 145,294,990 + 99,692,172 | 99.7 |
| pISLA-s1 | 5,606 | SRR23092357 | 1,809 (1%) | 47 | 99,692,172 | 99.8 |
| pMALL-c2 | 5,504 | ERR5979340 | 1510 (1%) | 48 | 81,576,380 | 99.8 |
| pPOLA-c1 | 6,075 | SRR13926770 | 1,415 (0%) | 59 | 8,866,062 | 100 |

[a] Plasmid names with suffix -c were assembled by contig extension; a sequence from an assembled contig was extended by rounds of repeated read mapping (see Methods). The suffix -s refers to plasmids assembled using metaplasmidspades (see Methods). The suffix -r in pTYRR-r1 indicates the initial contig was assembled from RNA-seq reads, and then extended by repeated rounds of read mapping to metagenomic reads.

[b] NCBI SRA read accessions are given. These were downloaded via the ENA (www.ebi.ac.uk/ena), trimmed and size filtered (see Methods), producing the number of reads shown in the Total Reads column.

[c] Number of reads mapped to the reference sequence at a maximum level of base differences shown in parentheses. In most cases a base difference tolerance of up to 1% was applied, while in some cases it could be set to zero % mismatches and the coverage still remained high. For pCABO-c1, values are given for both of the applied stringencies. The Geneious mapper (Geneious Prime version 2023.2.1) was set to custom sensitivity, fine tuning = none, minimum mapping quality = 40, only map paired reads which = both map, maximum mismatches per read = 1%, maximum ambiguity = 1.

[d] Average percentage identity of mapped reads to the reference sequence, as calculated by the Geneious mapper.
