# Supplementary material for: Global Distribution and Diversity of Haloarchaeal pL6-Family Plasmids
Source: Genes (Basel). 2024 Aug 26;15(9):1123. doi: 10.3390/genes15091123 (PMC11431627; doi:10.3390/genes15091123)
Supplement: Supplementary file 1 [file genes-15-01123-s001.zip › Table_S3_tetramers_v33.pdf]

Table S3. Absent or under-represented tetramers in pL6-plasmids[a]

	Plasmid[b]	Size (bp)	%G+C	Absent tetramers	Under-represented tetramers[c]
Previously published plasmids	pL6A	6,129	51.1	GGCC	CTAG^{3}
	pL6B	6,056	52.0	GGCC, CTAG	
	pBAJ9-6	6,213	53.0		GGCC^{4}[d], CTAG^{1}
	pLT53-7	7,045	50.5	GGCC	CTAG^{2}
	pLTMV-6	5,882	53.3	GGCC, CTAG	
Plasmids reconstructed in the current study	pCABO-c1	5,007	48.4	GGCC	CTAG^{4}
	pCABO-c10	5,219	51.4	GGCC, CTAG	
	pCABO-c2	5,889	53.7	GGCC, CTAG	
	pCABO-c6	5,864	53	GGCC, CTAG	
	pCABO-c9	6,930	49.3	GGCC	CTAG^{1}[e]
	pCABO-s1	6,206	47.8	GGCC, CTAG	
	**pCABO-s5**	5,017	**58.5**	-	
	pCOLO-c1	5,857	53.9	GGCC, CTAG	
	pHILL-c1	6,187	52.4	GGCC, CTAG	
	pHILL-c2	7,625	51.5	GGCC	
	pISLA-c6	5,881	53.6	GGCC, CTAG	
	**pISLA-s1**	5,606	**64.0**	TTAA	
	pMALL-c2	5,504	52.6	GGCC, CTAG	
	pPOLA-c1	6,075	48.1	GGCC	CTAG^{2}
	pTYRR-r1	5,844	51.0	GGCC	CTAG^{2}

[a]Tetramer searches were performed using the online tool (http://gscompare.ehu.eus/tools/oligo_frequencies/index.php)
[b]Grey shading indicates plasmids previously reported in [1]. Bold type denotes the high GC plasmids.
[c]Under-represented tetramers have a superscript number for the tetramer count.
[d]Four GGCC motifs occur within two closely spaced repeats found in the accessory gene region of this plasmid.
[e]The single CTAG site occurs in a foreign gene (SAM-dependent methyltransferase)

Reference

1. Dyall-Smith, M.; Pfeiffer, F. The PL6-family plasmids of *Haloquadratum* are virus-related. *Front. Microbiol.* **2018**, *9*, 1070.
